# Fine mapping of a QTL on bovine chromosome 6 using imputed full sequence data suggests a key role for the *group*-*specific component* (*GC*) gene in clinical mastitis and milk production

**DOI:** 10.1186/s12711-016-0257-2

**Published:** 2016-10-19

**Authors:** Hanne Gro Olsen, Tim Martin Knutsen, Anna M. Lewandowska-Sabat, Harald Grove, Torfinn Nome, Morten Svendsen, Mariann Arnyasi, Marte Sodeland, Kristil K. Sundsaasen, Sandra Rinne Dahl, Bjørg Heringstad, Hanne H. Hansen, Ingrid Olsaker, Matthew Peter Kent, Sigbjørn Lien

**Affiliations:** 1 Centre for Integrative Genetics (CIGENE), Department of Animal and Aquacultural Sciences, Norwegian University of Life Sciences, PO Box 5003, 1432 Ås, Norway; 2 Department of Basic Sciences and Aquatic Medicine, Norwegian University of Life Sciences, Oslo, Norway; 3 Geno Breeding and AI Association, 1432 Ås, Norway; 4 Institute of Marine Research, Flødevigen, 4817 His, Norway; 5 Department of Natural Sciences, Faculty of Engineering and Science, University of Agder, PO Box 422, 4604 Kristiansand, Norway; 6 Hormone Laboratory, Department of Medical Biochemistry, Oslo University Hospital, Oslo, Norway

## Abstract

**Background:**

Clinical mastitis is an inflammation of the mammary gland and causes significant costs to dairy production. It is unfavourably genetically correlated to milk production, and, thus, knowledge of the mechanisms that underlie these traits would be valuable to improve both of them simultaneously through breeding. A quantitative trait locus (QTL) that affects both clinical mastitis and milk production has recently been fine-mapped to around 89 Mb on bovine chromosome 6 (BTA6), but identification of the gene that underlies this QTL was not possible due to the strong linkage disequilibrium between single nucleotide polymorphisms (SNPs) within this region. Our aim was to identify the gene and, if possible, the causal polymorphism(s) responsible for this QTL through association analysis of high-density SNPs and imputed full sequence data in combination with analyses of transcript and protein levels of the identified candidate gene.

**Results:**

Associations between SNPs and the studied traits were strongest for SNPs that were located within and immediately upstream of the *group*-*specific component* (*GC*) gene. This gene encodes the vitamin D-binding protein (DBP) and has multiple roles in immune defense and milk production. A 12-kb duplication that was identified downstream of this gene covered its last exon and segregated with the QTL allele that is associated with increased mastitis susceptibility and milk production. However, analyses of *GC* mRNA levels on the available samples revealed no differences in expression between animals having or lacking this duplication. Moreover, we detected no differences in the concentrations of DBP and its ligand vitamin D between the animals with different *GC* genotypes that were available for this study.

**Conclusions:**

Our results suggest *GC* as the gene that underlies the QTL for clinical mastitis and milk production. However, since only healthy animals were sampled for transcription and expression analyses, we could not draw any final conclusion on the absence of quantitative differences between animals with different genotypes. Future studies should investigate *GC* RNA expression and protein levels in cows with different genotypes during an infection.

**Electronic supplementary material:**

The online version of this article (doi:10.1186/s12711-016-0257-2) contains supplementary material, which is available to authorized users.

## Background

Clinical mastitis (CM) is an inflammation of the mammary gland, which is generally caused by the introduction and multiplication of microorganisms. The consequences of CM include reduced animal welfare, economic losses due to reduced milk yield and altered milk composition, increased costs due to veterinary treatment of diseased animals, increased use of antibiotics, and early culling of animals. Thus, there is a high demand for solutions that help reduce the incidence of mastitis. Genetic improvement of resistance to CM by traditional breeding is possible [1], in spite of its low heritability [2] and an unfavorable genetic correlation with milk production (ranging from 0.21 to 0.55 in Norwegian Red; [3]). Knowledge of the genetic mechanisms that are involved both in susceptibility to mastitis and in milk production would be valuable information for selection programs to break the genetic correlation between these traits and improve both traits simultaneously. One approach to gain such knowledge is to identify associations between high-density or sequence level single nucleotide polymorphisms (SNPs) and the traits in question.

A quantitative trait locus (QTL) that affects susceptibility to CM or somatic cell score, which is an indicator trait of CM, was reported at ~90 Mb on bovine chromosome 6 (BTA6) in Norwegian Red cattle (NR) [4, 5] and in several Holstein populations [6–8]. Several genes that are known to be involved in immune defense, such as those that encode the CXC chemokines and are located at around 90.6 Mb, and the *IGJ* gene at 87.8 Mb that encodes the immunoglobulin J polypeptide, have been suggested as candidates for susceptibility to mastitis [5, 7]. However, two recent publications suggested that the gene *group*-*specific component* (*GC*) at 88.7 Mb was responsible for both a QTL that affects CM [9] and a QTL for milk production [10]. In both studies, the high level of linkage disequilibrium (LD) within this QTL region limited the ability to identify the causal polymorphisms.

*Group*-*specific component* is a logical functional and positional candidate gene. It is mainly transcribed in the liver [11] and encodes the vitamin D-binding protein (DBP), which circulates in blood and has multiple important roles in the immune system and host defense, e.g. as the main carrier of vitamin D. DBP has also been suggested to have a direct role on milk production [10].

In this study, we performed association analyses on high-density SNP chips and imputed full sequence data in order to identify the gene, and if possible the polymorphism (or polymorphisms) on BTA6 behind the variation in susceptibility to CM and in milk production. We also studied splice variants and determined the transcript levels of the identified gene in liver samples, and analyzed the concentrations of DBP and its ligand in serum samples from individuals with different *GC* genotypes.

## Methods

### Biological samples

Three different sets of samples were used:

(A) Semen samples from Norwegian Red artificial insemination (AI) bulls for which phenotypic information was available for SNP genotyping (the number of bulls varied among analyses as described in the relevant sections), and semen samples from NR bulls and blood samples from NR cows for genome resequencing.
(B) Liver samples from 112 healthy NR animals, mostly bulls, from which DNA was extracted for SNP genotyping; 10 of these 112 individuals were selected for analysis of mRNA expression.
(C) Blood serum samples from 50 healthy NR cows, of which 21 were pregnant at the time of sampling, for analysis of protein expression and vitamin D levels.

### DNA and RNA isolation

DNA was extracted from semen and blood using standard protocols. Liver samples were collected *post*-*mortem* during two consecutive days at one specific abattoir at approximately the same time after culling of each individual. Slaughter started in the morning and all sampled animals were culled within 2.5 h. Data from the Norwegian Health Card system showed that all animals were healthy. All animals were between 512 and 628 days old at the time of the sampling. DNA from the liver samples was extracted (NucleoSpin Tissue kit, Macherey–Nagel, Hoerdt) and then used for genotyping with the SNP that was most strongly associated with CM and with the detected duplication.

Based on the genotypes for rs110675140, liver samples from ten unrelated bulls (five for each homozygous genotype) from randomly selected farms (in order to randomize the environmental effect) were selected and used for analysis of mRNA expression. RNA was isolated by using the QIAzol lysis reagent (Qiagen, Hilden, Germany) and on-column purification NucleoSpin RNA kit (Macherey–Nagel) with on-column DNase digestion according to the manufacturer’s instructions. RNA concentration and quality were measured with a NanoDrop 1000 spectrophotometer (Thermo Fisher Scientific, Wilmington) and Agilent RNA 6000 Nano LabChip on the 2100 Agilent BioAnalyzer (Agilent Technologies, Palo Alto), respectively. All samples had an RNA integrity number (RIN) between 7 and 8 and an OD A260/A280 ratio of approximately 2.

### Preparation of serum samples

Sampling of blood for serum analysis was performed by a certified veterinarian and thus conducted in agreement with the provisions enforced by the Norwegian Animal Research Authority. More details about the tested animals are in Additional file 1: Table S1. Blood was collected in 10 mL collection tubes (BD Vacutainer^®^ Plus Plastic Serum Tubes, Becton, Dickinson and Company, Franklin Lakes), using three tubes per animal. The samples were allowed to clot at room temperature for approximately 30 to 60 min, prior to centrifugation at 2000*g* (10 min, 4 °C). The serum was immediately transferred into a new tube and stored at −20 °C until analysis.

### Traits

Daughter yield deviations (DYD) for seven CM traits and five milk production traits were derived for AI bulls using solutions from the national genetic evaluation of the NR population that is carried out by GENO Breeding and AI Association.

In the routine genetic evaluation of CM, records on the veterinary treatments applied to treat CM are used to define seven binary traits, each of these indicating the occurrence of CM in one of seven time intervals. These time intervals include three periods during the first lactation: CM1 [−15 to 30 d postpartum (dpp)], CM2 (31 to 120 dpp) and CM3 (121 to 305 dpp), two periods during the second lactation: CM4 (−15 to 30 dpp) and CM5 (31 to 120 dpp); and two periods during the third lactation: CM6 (−15 to 30 dpp) and CM7 (31 to 120 dpp).

Five milk production traits, i.e., 305 d lactation yields for kg milk, kg protein, kg fat, protein percentage and fat percentage, were based on monthly test-day records of milk yield and bimonthly records of kg fat and kg protein. Animal repeatability models that were based on data from the first to third lactation were used for the genetic evaluation of milk production traits.

The number of bulls with trait data varied for each trait, especially in the case of CM traits because many cows were culled before they reached the last period of the last lactation. In addition, selection of animals for genotyping was performed to fit the needs of several studies, and hence the number of bulls with both trait and genotype data varied among analyses as described below.

### Construction of a high-density SNP dataset for BTA6

Genotypes from the Affymetrix 25K SNP array (Affymetrix, Santa Clara), the Illumina BovineSNP50 BeadChip (54K) and the Illumina BovineHD Genotyping BeadChip (777K) (Illumina, San Diego) were combined to produce a dataset for BTA6 that consisted of 26,737 SNPs. First, 2552 NR AI bulls were genotyped on the 25K chip. Then, 1575 NR bulls were genotyped on the 54K chip with 536 of these 1575 animals already genotyped on the 25K chip. Finally, 384 of the 1575 bulls were genotyped on the 777K chip. The three datasets were filtered to remove SNPs with a minor allele frequency lower than 0.05. Map positions were based on the bovine reference genome assembly (UMD 3.1; [12]). SNPs on unplaced scaffolds and on sex chromosomes were discarded. The 25K dataset was imputed to 54K and then the combined 54K dataset was imputed to 777K. All imputations and phasing of data were completed using BEAGLE v3.3.1 [13]. Phasing information was used to identify double recombinants, i.e. a heterozygous genotype with a phase that differed from the phase on both sides. Such genotypes were either corrected (for instance from AG to GA) or removed. All five milk traits and seven CM traits were analyzed for these 26,737 SNPs on BTA6. There were 3090 bulls with genotype data and DYD information for the five milk traits, CM1 and CM2, and this number decreased with later CM periods to 2874 bulls for CM7.

### Genome sequencing, alignment of sequence reads and variant calling

Whole-genome sequencing data were obtained for 147 animals at three different time points in batches of 5, 16 and 126 animals. A first batch of five bulls were sequenced by FASTERIS (Switzerland) on an Illumina Genome Analyzer GAIIx instrument (Illumina, San Diego, CA, USA) with 2 × 108 bp paired end reads. The TruSeq SBS V2-GA kit was used to prepare libraries and the FASTX-toolkit v0.0.13 (Hannon Lab, FASTX-Toolkit, 0.0.13 ed2010) for adaptor- and quality-trimming of raw reads in FASTQ-format. An average sequence coverage of ~4× and 10× was obtained for three and two bulls, respectively. A second batch of 16 elite bulls were sequenced by AROS (Denmark) on a HiSeq 2000 platform according to the manufacturer’s protocols. Samples were prepared for paired-end sequencing (2 × 100 bp) using the TruSeq kit and sequenced with the Illumina V3 kit (Illumina, San Diego) to generate an average coverage of 10×. Finally, the third batch that included 96 NR elite bulls and 31 NR cows were sequenced by the Norwegian Sequencing Centre (http://www.sequencing.uio.no) on a HiSeq 2500 platform according to the manufacturer’s protocols. Samples were prepared for paired-end sequencing (2 × 125 bp) by using the TruSeq DNA PCR-free library preparation kits and sequenced with the Illumina V4 kit (Illumina, San Diego) to generate an average coverage of 9×. Average sequencing coverage in a 1-Mb region that included the most significant markers and the duplication agreed with the genome-wide average coverage.

The quality of the Illumina reads was checked with fastQC [14]. Adaptors were trimmed as described above but no quality trimming of the Illumina reads was done before alignment, as recommended when the read quality is sufficiently high (all samples had mean PHRED quality scores above 30 throughout the whole length of the reads). All reads were aligned against the UMD 3.1 bovine genome assembly using BWA-mem version 0.7.10 [15] and then PCR duplicates were sorted and marked and the resulting SAM files were indexed using SAMtools version 1.2 [16]. Between 98.7 and 99.6 % of the reads were mapped to the reference genome, including all chromosomes and unplaced scaffolds. Variant calling was done with FreeBayes version 1.0.2 [17], with a minimum total read coverage of 10, a minimum PHRED-based mapping score of 20 and a minimum PHRED-based base quality of 20. All called variants were filtered prior to phasing by using bcftools version 1.2, which is a part of the SAMtools program suite [16]. The applied filters were run with the following bcftools command: bcftools filter --exclude “QUAL < 30 | NUMALT > = 2 | SAF == 0 | SAR == 0 | DP < 10 | DP > 2500 | MAF < 0.01” --SnpGap 4 --IndelGap 10, which will exclude all variants that do not fulfill one of the following criteria: (1) an overall quality (QUAL) score less than 30, (2) a total depth with less than 10 or more than 2500 reads, (3) if no read was observed on either the forward or reverse strand, (4) if there was more than one alternate allele, (5) if the minor allele frequency was lower than 1 %, (6) if a SNP was closer than 4 bp to an INDEL and (7) for any two INDEL that were within 10 bp of each other, the lower quality INDEL was removed. These filtering criteria are similar to those used in the 1000 bull genome project (http://www.1000bullgenomes.com/), and were chosen to minimize the number of false positive variants, while sacrificing some degree of variant calling sensitivity.

### Imputation to full sequence

Genotypes of the called variants were refined and phased using Beagle version 4.1 [13]. The initial data input to Beagle was the genotype likelihood of each genotype reported by Freebayes. The resulting phased dataset was then used as a reference panel for imputing all previously mentioned 3090 animals to full sequence, ending up with a total of 3096 animals with full sequence data and phenotypes for CM1. Beagle 4.1 was run with the window parameter set to 100,000 SNPs and a window overlap set to 10,000 SNPs. Effective population size was set to 200 and remaining parameters were set to default values. Sequence data will be submitted to the European Nucleotide Archive.

### Single marker association studies

Single marker analyses were first performed for all 12 traits and the 26,737 SNPs on BTA6 from the high-density BTA6 dataset, and then for the six traits that were significant in the first analyses and 65,156 markers (SNPs and indels) between 84 and 94 Mb on BTA6.

The initial association analysis of the high-density dataset was performed with the ASREML package version 2.0 [18]. The model that was fitted to the information on performance for each trait—marker combination was:

$${\mathbf{DYD}} = \mathbf{1}\mu + {\mathbf{X}}{\text{b}} + {\mathbf{Za}} + {\mathbf{e}},$$

where **DYD** is the vector of bull performances weighed by the number of daughters, **1** is a vector of ones, *μ* is the overall mean, **X** is a vector of SNP genotypes coded as 0, 1, or 2 depending on the number of copies of the first allele, b is the fixed effect of the marker, **Z** is an incidence matrix relating phenotypes to the corresponding random polygenic effects, **a** is a vector of random polygenic effects, and **e** is a vector of residual effects. Genetic and residual variances were estimated from the data. **a** was assumed to follow a normal distribution ~$N\left( {0,{\mathbf{A}}\sigma_{{\mathbf{A}}}^{2} } \right)$ where **A** is the relationship matrix derived from the pedigree, and $\sigma_{{\mathbf{A}}}^{2}$ is the additive genetic variance. **e** was assumed to follow a normal distribution ~$N\left( {0,{\mathbf{I}}\sigma_{{\mathbf{e}}}^{2} } \right)$ where $\sigma_{{\mathbf{e}}}^{2}$ is the residual variance. Association analysis was performed for each individual marker, and then the p value for the marker effect was calculated with the R function pf(). The chromosomal Bonferroni corrected significance threshold for the HD map was set at [−log10(0.001/26,737)] = 7.43, corresponding to a nominal type I error rate of 0.001 after Bonferroni correction for 26,737 tests.

For the association analysis of the imputed full sequence data, we used the GCTA package (genome-wide complex trait analysis) with the mixed linear model based association analysis (MLMA) option [19] for computational efficiency. The model fitted with GCTA MLMA is as follows:

$${\mathbf{DYD}} = \mathbf{1}\mu + {\mathbf{X}}{\text{b}} + {\mathbf{Zg}} + {\mathbf{e}}.$$

The model description is similar to that used for the ASReml analysis, except that **DYD** is now an unweighted vector of bull performances, and **g** is the random polygenic effect, i.e. the accumulated effect of all SNPs as captured by the genetic relationship matrix which was estimated by GCTA as described by Yang et al. [19]. The genetic variance is estimated based on a null model without the marker effect, and then used in the alternative model to test for associations between each of the SNPs and the trait [19].

### Correction for the most significant QTL

In order to determine if more than one QTL segregated for any of the six tested traits (i.e., CM1, CM4, CM6, kg milk, kg protein and kg fat) the effect of the most significant marker for CM1 from the single marker analyses was corrected for by including this marker as a cofactor using the mlma-no-adj-covar option in GCTA [19].

### Selection of animals with different QTL genotypes

In a preliminary step using only a subset of the sequence-level markers, the most significant SNP for CM1 was located in intron 1 of *GC*, i.e. rs110675140 at 88,728,121 bp (unpublished results). Allele A of rs110675140 was associated with increased susceptibility to mastitis and also with higher milk yield than allele G. Since the imputed full sequence data were not available when the analyses of structural variations, mRNA and protein analyses were performed, rs110675140 was used to select animals that carried the appropriate genotypes for these analyses. An LD (r^2^) of 0.84 was found between rs110675140 and the most significant marker for CM1 in the full sequence analyses using the Haploview 4.2 software [20].

### LD measurements

Pair-wise LD measurements (r^2^) were estimated for all pairs of sequence-level markers using the Haploview 4.2 software [20] on phased data. Haplotypes were defined by Haploview according to the confidence intervals strategy [21] or the four gamete rule [22].

### Structural variations

Raw data from three bulls that were homozygous AA for rs110675140 and five bulls that were homozygous GG were merged into one BAM file that included all three AA bulls and one BAM file that included all five GG bulls using SAMtools [16], which were used to identify larger segregating structural variations in the genome by the CNV-seq software [23]. Significant results were initially examined and visually inspected using the integrative genomics viewer software (IGV [24]), and confirmed by PCR on a larger number of animals. PCR primers to verify the duplication were designed using Primer3 software ver. 4.0.0 [25]. One set of primers (5′-GTCTTTCGAACTGCAGGACT-3′ and 5′-TCCCATTGAGCTAAGCCTGG-3′) was designed to amplify a 221-bp region that spanned from the end of one copy to the start of the next copy, while the second primer pair (5′-GTCTTTCGAACTGCAGGACT-3′ and 5′-ACTCATGTGTGTTGCCCTCT-3′) was designed to amplify a fragment of 370 bp in a non-duplicated region as an internal control of the PCR reaction. The PCR reaction contained 1 U of Taq polymerase, 10× Taq reaction buffer, 1 mM of MgCl_2_, 0.25 mM of each dNTP, 0.2 pmol/µL primer “Internal control primer pair”, 0.25 pmol/µL of “duplication primer pair” and 20–40 ng of template DNA. The reaction conditions included an initial denaturation step at 95 °C for 5 min, followed by 35 cycles at 95 °C for 30 s, 58 °C for 30 s and 72 °C for 1 min, and a final extension at 72 °C for 10 min. The completed multiplex PCR reaction was electrophoresed on a 1 % agarose gel. For animals without the duplication, only the internal control fragment (370 bp) was amplified, while for animals with the duplication in either the homozygous or heterozygous form, both the duplication fragment (221 bp) and the internal control band were amplified. The PCR was run on 45 NR AI bulls and on 112 liver samples with a known genotype at rs110675140. Of these 112 animals, 23, 47 and 42 animals carried the AA, GG and AG genotype, respectively at rs110675140.

### Investigations to identify the putative function of the QTL

Several approaches were used to identify the underlying causal polymorphism of the QTL. We first checked if the significant markers were conserved in mammals using the Ensembl database (http://www.ensembl.org). Next, the bovine sequence was blasted (http://blast.ncbi.nlm.nih.gov/Blast.cgi) against the human sequence to identify possible functional sites (e.g. binding sites for transcription factors, sites affecting alternative splicing, etc.) using all available tracks for expression and regulation in the UCSC Genome Browser (https://genome.ucsc.edu). Finally, putative transcription factor binding sites in the bovine sequence were identified using the JASPAR database (http://jaspar.genereg.net/) and Match public version 1.0 (http://www.gene-regulation.com/cgi-bin/pub/programs/match/bin/match.cgi).

### Identification and quantification of putative splice variants by RNAseq

Differential expression of alternatively spliced variants among ten bulls with different genotypes (five AA and five GG bulls at rs110675140) was analyzed on mRNA isolated from liver samples. These ten bulls were selected based on pedigree information to be as unrelated as possible, and originated from random farms in order to randomize environmental effects. Ten libraries representing liver samples from the five AA and five GG bulls were prepared for sequencing using an Illumina TruSeq stranded mRNA LT Set-A kit (Illumina, California, USA) with the following slight modifications to the manufacturer’s protocol: (i) using 2 µg input RNA, (ii) incubation for 25 s (vs. 8 min) at the “elute, fragment and prime” step, and (iii) using a cDNA:read ratio of 1:0.7 ratio (vs. 1:1) (Agencourt AMPure XP Beads; Beckman Coulter, California) after adapter ligation and PCR step. Libraries were validated by quantification using a Qubit^®^ 2.0 Fluorometer and the Qubit^®^ dsDNA BR Assay kit (Invitrogen™, Thermo Fisher, Massachusetts) and by size analysis using a 2100 BioAnalyzer system with the DNA High Sensitivity kit (Agilent Technologies, California). The mean concentration and length for the libraries were 40 ng/µL and 700 bp respectively. For normalization, the libraries were diluted to 10 nM and pooled in equal ratios. Sequencing was performed on a MiSeq (Illumina, California) with the 600 cycles MiSeq reagent kit v3 in a paired-end mode.

All raw data of reads were formatted as fastq files. Before mapping, Illumina adaptors were removed and the sequences were quality-trimmed using cutadapt [26]. Cutadapt was set to cut adaptors with a minimum overlap length of 8 and low-quality 3′ ends were removed by setting a quality threshold of 20 (phred quality + 33). An index of the bovine UMD 3.1 reference genome was built and reads were aligned to the reference genome using STAR version 2.3.1 [27]. Sorting, indexing and conversion to the BAM file format (the compressed binary version of a SAM file) of the resulting SAM files (sequence alignment/map format) were completed by using SAMtools version 0.1.19 [16]. Read alignments were assembled to transcripts (NCBI *Bos taurus* Annotation Release 104) using Cufflinks version 2.2.1 [28] with a minimum isoform fraction set to 1 %. Finally, the R package DEXSeq [29] was used to test for differential exon usage between GG and AA animals.

### Quantification of known alternative spliced variants of the *GC* gene using RT-qPCR

Results from the RNAseq analyses were confirmed by reverse transcription quantitative PCR (RT-qPCR) on samples of total liver RNA from ten animals, which were the same as those used for RNAseq. The samples represent technical replicates, i.e. RNA for RT-qPCR and RNA for RNAseq were isolated in parallel. Five hundred nanogram of total liver RNA was reverse-transcribed using the Tetro cDNA synthesis kit (Nordic BioSite, Oslo), the cDNA was then diluted and 6.25 ng was used for qPCR. qPCR was performed in 20 µL reaction volumes and in triplicate per sample using Express SYBR GreenER SuperMix with premixed ROX (Invitrogen) according to the manufacturer’s recommendations. Transcript levels were analyzed using a 7900HT Fast Real-Time PCR System (Applied Biosystems) and the following program: 95 °C for 2 min, 40 cycles at 95 °C for 15 s and 60 °C for 1 min, and the melting curve analyses were applied. Primers specific for the mRNA reference sequence of the *Bos taurus GC* gene (RefSeq; NM_001035380), the predicted transcript variant 1 (X1; XM_005208092.1) and the predicted transcript variant 2 (X2; XR_234992.1) were designed using Primer3 ver. 0.4.0 [25]. Primer sequences are in Additional file 2: Table S2. The efficiency of all primer pairs was tested by template dilution series and reached 81 % for the primers specific to the X2 variant and ranged from 90 to 100 % for primers specific to the RefSeq and X1 variants. Negative controls with no added template were included for all primer pairs (no template control), and no RT control reactions for each sample and each primer pair were run in qPCR in order to check for genomic DNA contamination. Based on the literature [30], peptidylprolyl isomerase A (PPIA) was used as reference gene (control). An initial analysis of the RT-qPCR data was performed using RQ Manager 1.2 (Applied Biosystems) and a standard deviation of less than or equal to 0.3 per triplicate was accepted. The ΔCt method was used for the statistical analysis of the RT-qPCR data, i.e. ΔCt = Ct_target_ − Ct_control_, and normalized gene expression was calculated as 2^(−ΔCt)^. The differences between transcript levels in animals with different genotypes, and between the three transcript variants, were tested using a t test.

### Protein concentrations and ligand binding

Serum concentrations of several proteins in 50 NR cows (15 with genotype AA and 35 with genotype GG at rs110675140) were estimated as follows: DBP concentrations were determined by Vitas-Analytical Services (Oslo, Norway) using an enzyme-linked immunosorbent assay (ELISA kit; Antibodies-online GmbH, Aachen) according to the manufacturer’s instructions. Absorbance was detected at 450 nm. Albumin (ALB) concentrations were determined by The Central Clinical Laboratory (Sentralaboratoriet, NMBU School of Veterinary Medicine, Oslo) with the bromocresol green (BCG) dye-binding method (Advia 1800 Chemistry System, Siemens Healthcare, Germany). Serum albumin quantitatively binds to BCG to form an albumin-BCG complex that was measured as an endpoint reaction at 596 nm. 25-Hydroxy-vitamin D [25(OH)D2 and 25(OH)D3] concentrations were determined by applying a liquid chromatography–tandem mass spectrometry (LC–MS/MS) method developed at the Hormone laboratory (Oslo University Hospital). More details on these procedures are in Additional file 3. Free, bioavailable, ALB- and DBP-bound 25(OH)D2, 25(OH)D3 and total 25(OH)D [a sum of 25(OH)D2 and 25(OH)D3] were calculated using modified equations previously described in [31, 32], based on the serum concentrations of 25(OH)D, DBP and ALB. Briefly, this method defines the bioavailable vitamin D as the fraction that is both free and ALB-bound, i.e. not bound to DBP. For this calculation, the human affinity-binding constants for 25(OH)D interaction with DBP and ALB described in [33] were used. General linear model (GLM) analyses in SAS 9.2 (SAS Institute Inc., Cary, NC) were used to determine possible effects of the rs110675140 genotype on the concentrations of DBP, ALB, 25(OH)D2, 25(OH)D3 and total 25(OH)D; free 25(OH)D2, 25(OH)D3 and total 25(OH)D; bioavailable (i.e., free and ALB-bound) 25(OH)D2, 25(OH)D3 and total 25(OH)D; DBP-bound total 25(OH)D; and ALB-bound total 25(OH)D in the serum. The model included three fixed effects, i.e. rs110675140 genotype (two classes, AA and GG), lactation number (five classes), and stage of lactation at the time of sampling (months after calving, six classes) as explanatory variables. The general linear model analyses were performed with and without nine heifers that had not had their first calf at the time of sampling.

## Results and discussion

### Association analyses of mastitis and milk production traits with the high-density SNP set on BTA6

**Fig. 1:**
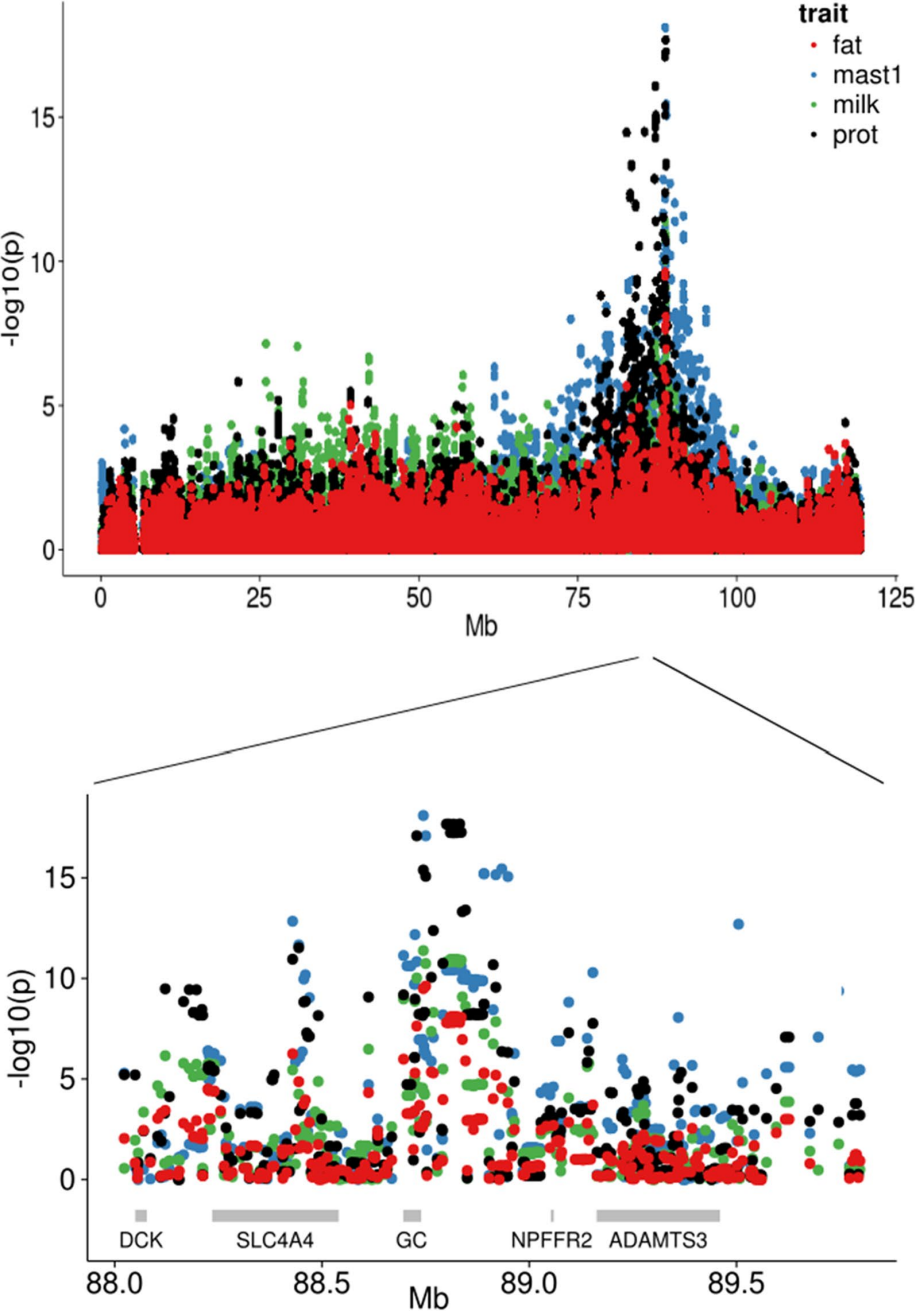
Results for single-marker association analysis of CM1, kg milk, kg protein and kg fat on the high-density map for BTA6. *Top* Results for all tested SNPs on BTA6. −log_10_(p value) on the *y-axis* and marker position in Mb on the *x-axis*. *Bottom* Zoom on the region between 88.0 and 89.7 Mb. Gene positions are indicated with *grey rectangles*

Single marker association analyses were performed for all seven CM traits and five milk production traits and ~27,000 SNPs on BTA6. Highly significant results were detected for CM1, CM4, CM6, kg milk, kg protein, kg fat and protein percentage in a region between ~88.7 and 88.9 Mb on BTA6 (Table 1). The strongest association was observed between CM1 and rs110300280 at 88,744,593 bp. This marker was also the most significant marker for CM4 and kg milk. No significant associations were found for CM2, CM3, CM5 and CM7 on BTA6. As examples, results for CM1, kg milk, kg protein and kg fat are in Fig. 1, and complete results for all traits and SNPs are in Additional file 4: Table S3.

**Table 1:** Results from association analyses for the SNPs on BTA6 from the Illumina BovineHD BeadChip

Trait^a^	SNP	bp	−log(p)	Frequency	Effect	SE	A1	A2
CM1	rs110300280	88,744,593	18.1	0.37	0.007	0.0008	A	G
CM4	rs110300280	88,744,593	11.9	0.37	0.006	0.0008	A	G
CM6	rs109066341	88,890,502	11.5	0.41	0.007	0.001	G	A
kg milk	rs110300280	88,744,593	11.4	0.37	46.07	6.619	A	G
kg protein	rs134055603	88,832,335	17.7	0.42	1.65	0.187	A	G
kg fat	rs110679276	88,750,266	9.6	0.38	1.70	0.2669	A	G

For each trait, the most significant SNP for the QTL that was detected close to the *GC* gene is shown with its position in base pairs (bp), negative logarithm of the p value [−log(p)], minor allele frequency (Freq), allele substitution effect for the first allele of the SNP (Effect), standard error of the SNP effect (SE), and alleles

^a^CM1, CM4 and CM6: clinical mastitis in the period from −15 to 30 days postpartum in the first, second and third lactations, respectively. Performance data for kg milk, kg protein and kg fat refers to 305 d lactation yields

The most significant SNP in this analysis, rs110300280, was located approximately 5400 bp 5′ from the transcription start site of the *GC* gene. *GC* is transcribed on the reverse strand between 88.74 and 88.69 Mb (according to the UMD 3.1 assembly of the bovine genome) and is the only known gene in close vicinity of this QTL position. The closest genes on either side are *SLC4A4* [*solute carrier family 4 (sodium bicarbonate cotransporter), member 4*] at ~88.2–88.5 Mb and *NPFFR2* (*neuropeptide FF receptor 2*) at ~89 Mb, but the significance levels of all traits were markedly reduced for SNPs within and closer to these genes, and hence our analyses indicate *GC* as the strongest positional candidate gene for the QTL.

### Association analysis of mastitis and milk production on full sequence data

The traits CM1, CM4, CM6, kg milk, kg protein and kg fat were reanalyzed for a total of 65,156 SNPs between 84 and 94 Mb on BTA6 that were detected by whole-genome resequencing. All detected sequence level variations within the *GC* gene as well as within a flanking region of ~5 Mb on each side of the gene were analyzed in order to verify that *GC* was the causal gene and, if possible, identify the causal variations within this gene. For computational reasons, the analyses were performed with a different software than in the previous step. The p values were lower in this step than in the previous one, most likely due to differences in the algorithms used.

**Fig. 2:**
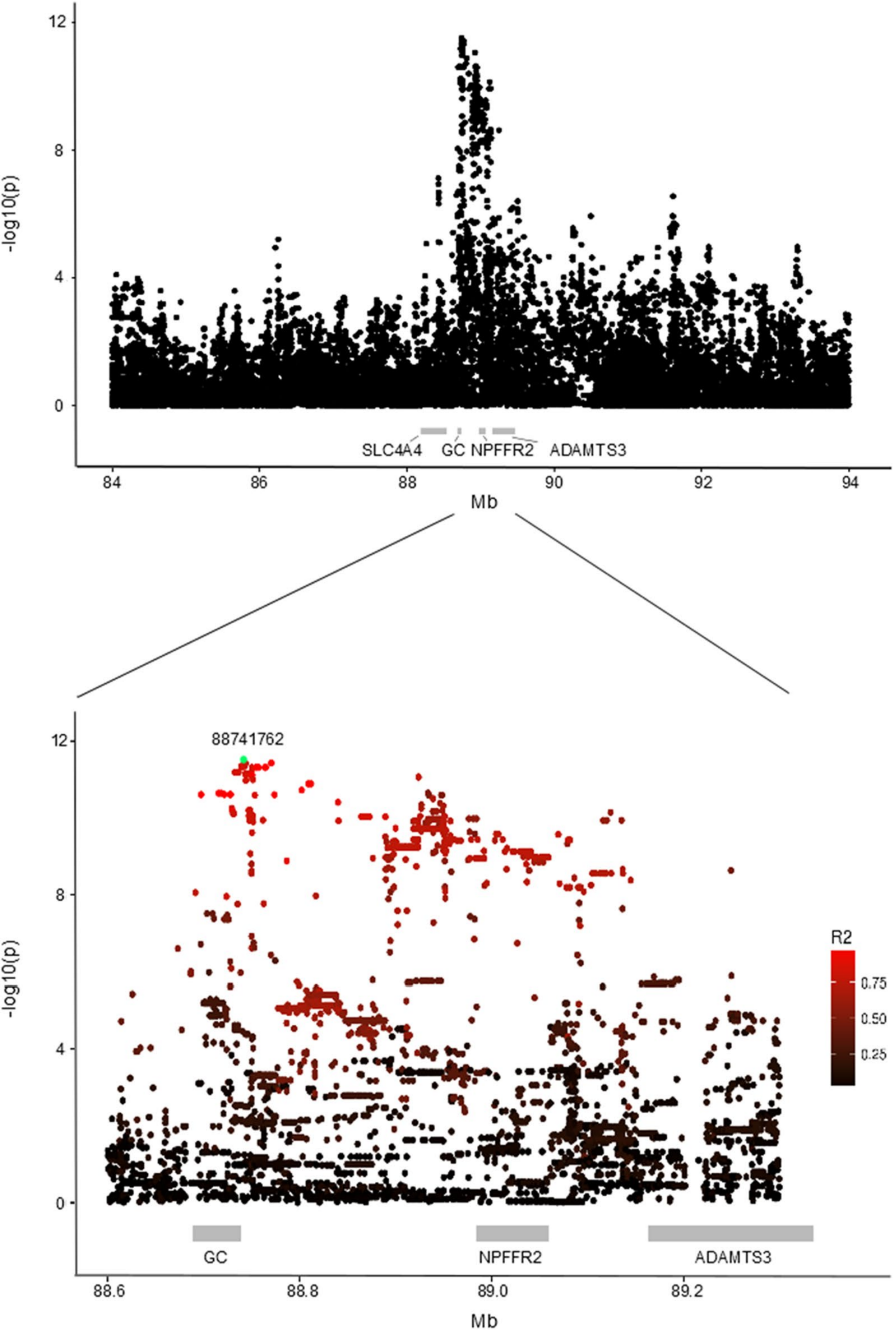
Results for single-marker association analyses of CM1 using imputed full sequence data. *Top* Results for all tested SNPs. *Bottom* Zoom on the region between 88.6 and 89.2 Mb. Gene positions are indicated with *grey rectangles*. The most significant marker, rs110483492 at 88,741,762 bp, is highlighted in *green*

For all traits tested, a series of SNPs with very similar p values were detected within and close to *GC* (Fig. 2; Table 2). They were all located either in introns of the *GC* gene or immediately before the gene on the 5′ end, and showed markedly higher significance levels than all SNPs in the coding regions. The ranking of the SNPs differed somewhat among the traits (Table 2). CM1 and kg milk were most strongly associated to rs110483492 at 88,741,762 bp [−log_10_(p value) = 11.52 and 12.75, respectively], which is located ~2.5 kb 5′ of the transcription start site. The G allele of rs110483492 was associated to higher mastitis incidence and higher milk yield than the A allele, and showed a frequency of ~34 % among the genotyped bulls. Nearly similar significant p values were found for several other SNPs in intron 1 of *GC* and immediately upstream of the gene. kg fat was most significantly associated to rs381872595 [−log_10_(p value) = 7.76], an indel at ~88,743,767 bp that was also among the most significant markers for CM1 and kg milk. kg protein, CM4 and CM6 showed the strongest associations with markers at ~88.9 Mb and with markers within and immediately close to *GC*. Results for all traits and markers are in Additional file 5: Table S4. However, as shown in Fig. 2, the significance levels decreased only moderately from *GC* towards *ADAMTS3*, and the possibility that the QTL effect is due to polymorphisms closer to *NPFFR2* or *ADAMTS3* could not be completely ruled out. On the other hand, none of the markers in the coding or promoter sequences of these genes were among the most significant.

**Table 2:** Results from association analyses of SNPs in the candidate gene region

Trait^a^	SNP	bp	−log(p)	Freq	Effect	SE	A1	A2
CM1	rs110483492	88,741,762	11.5	0.34	0.007	0.001	G	A
CM4	rs208489948	88,808,884	7.0	0.34	0.005	0.001	T	C
CM6	rs109133718	89,069,508	9.1	0.36	0.008	0.001	C	T
kg milk	rs110483492	88,741,762	12.8	0.34	60.8	8.26	G	A
kg protein	rs385796667	88,804,540	12.8	0.39	1.73	0.23	G	A
kg fat	rs381872595	88,743,767	7.8	0.36	1.91	0.34	Del	Ins

The most significant SNP for each of the traits is shown with its position in base pairs (bp), negative logarithm of the p value [−log(p)], minor allele frequency (Freq), allele substitution effect for the first allele of the SNP (Effect), standard error of the SNP effect (SE), and alleles

^a^CM1, CM4 and CM6: clinical mastitis in the period from −15 to 30 days postpartum in first, second and third lactation, respectively

The more telomeric positions of the most significant markers for kg protein, CM4 and CM6 could suggest that additional polymorphisms affected these traits. However, when the effect of rs110483492 at 88,741,762 bp was corrected for, no markers remained significant for any of the six traits. Hence, all significant markers are most likely in strong LD with the same causal polymorphism.

The use of full sequence data did not refine the QTL region much more compared to the first step. However, since the sequence data include all the variants that are present in a larger region, other genes than *GC* could be excluded as causal. Moreover, the analysis suggests that the underlying causal variation has a regulatory role since no variants within the coding sequences were among the most significant ones.

### LD structure

LD analyses revealed a relative complex LD structure, which reflects the history of the NR breed. Highly significant SNPs were located in a larger region that spanned the entire *GC* gene and several kb from its 5′ end, and were dispersed in-between other SNPs that had much lower significance levels. All the SNPs with a p value lower than 1*e−11 for CM1 were in high LD with each other, most often with an r^2^ higher than 0.95. One unexpected exception was the most significant SNP for CM1 at 88,741,762 bp, which showed an r^2^ with the other top SNPs of ~0.84. The top-ranking SNPs were distributed across a large number of haplotype blocks, and most blocks contained SNPs with highly variable significance levels. For CM1, most of the SNPs with a p value lower than 1*e−12 were in three blocks that covered the region between 88,739,045 and 88,744,985 bp, i.e., a region that spanned from the beginning of intron 1 to ~5 kb from the transcription start site. For each of these SNPs, the allele that was associated with the highest mastitis incidence and milk yield was unique to a haplotype with a frequency of 37 %, which is in agreement with the frequency of the individual alleles.

Such an LD pattern that consists of a high LD between rather distant SNPs, which are interspersed in-between many other SNPs with a much lower LD is typical for most commercial cattle breeds that have undergone a rapid recent decrease in effective population size from a large ancestral population due to selection and breed formation. The long-ranging LD is most likely due to the presence of some long haplotypes that are the result of the recent extensive use of AI bulls and that have not been broken apart by recombination, in a background of much shorter haplotypes that reflect the large ancestral population. The size and the combinational effects of such haplotypes limit our ability to fine map and precisely identify the causal polymorphism(s) that underlie the QTL based only on association mapping results.

### Structural variation

**Fig. 3:**
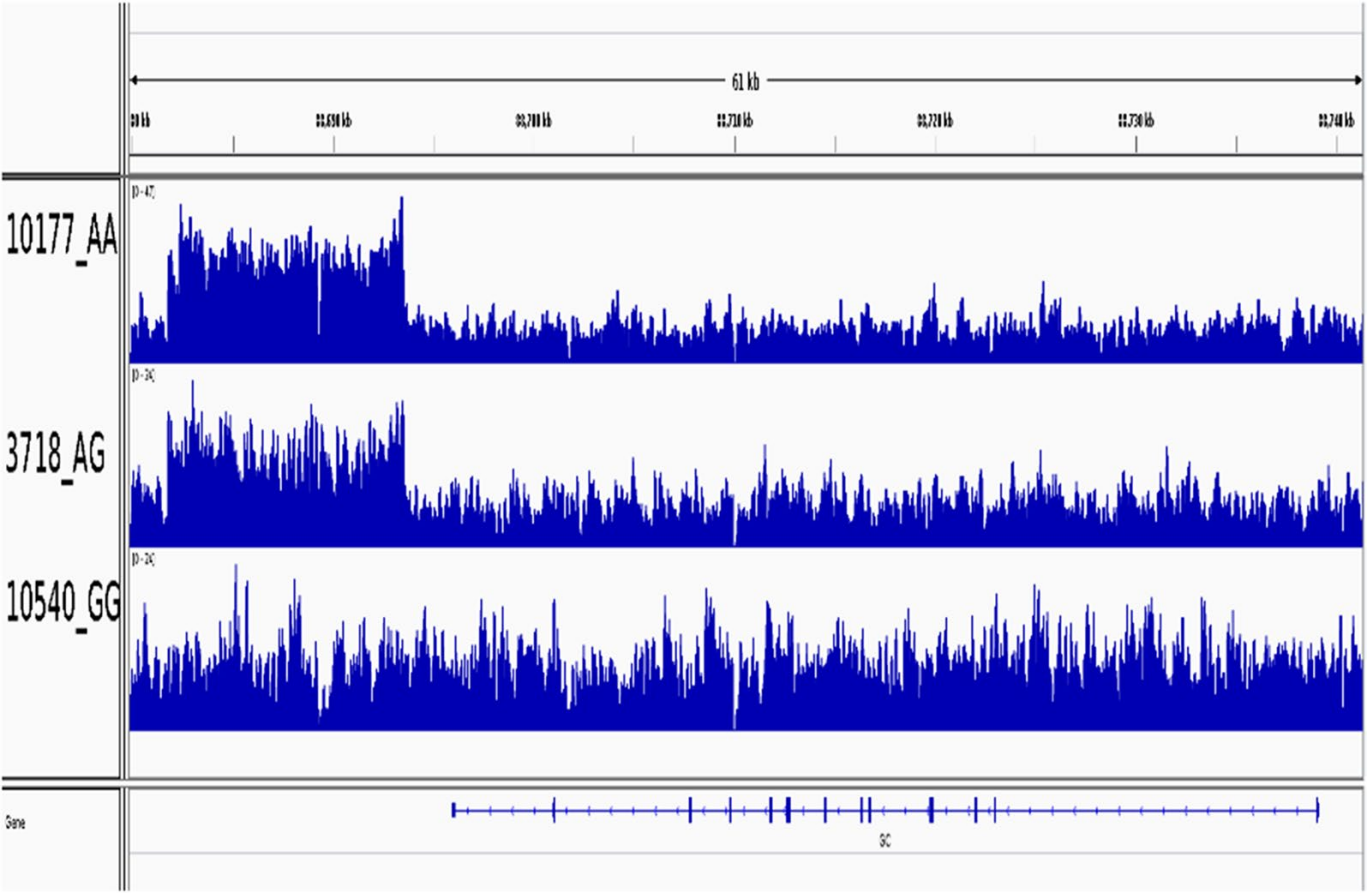
Visualization of the duplication. The first animal, 10177, is homozygous AA at rs110675140 and shows an increased read depth in a region 3′ of *GC*. This indicates that the region is duplicated. Moreover, the duplication is found in both haplotypes since the read depth is doubled compared to the surrounding regions. The second animal, 3718, is heterozygous GA, and has an intermediate read depth. This indicates that the duplication is present in one haplotype only (i.e., the one that also carries allele A). The last animal, 10540, is homozygous GG at rs110675140, and has a normal read depth, which indicates that there is no duplication

A search for larger structural variants in resequencing data from 21 NR sires revealed a marked increase in read depth for a 11,781-bp region between 88,693,550 and 88,681,769 bp (i.e., 2.4 kb 3′ of the last ordinary exon of *GC*, see below) for animals that are homozygous AA at rs110675140 (Fig. 3). This increase was not present in the homozygous GG animals, while for the heterozygous GA animals an intermediate increase in read depth was observed (Fig. 3). This indicates that the sequence is duplicated in the haplotypes that carry the A allele, but not in those that carry the G allele. The orientation of the paired end reads that span the breakpoint of the duplication suggested that the two copies of the sequence were orientated as a tandem duplication.

**Fig. 4:**
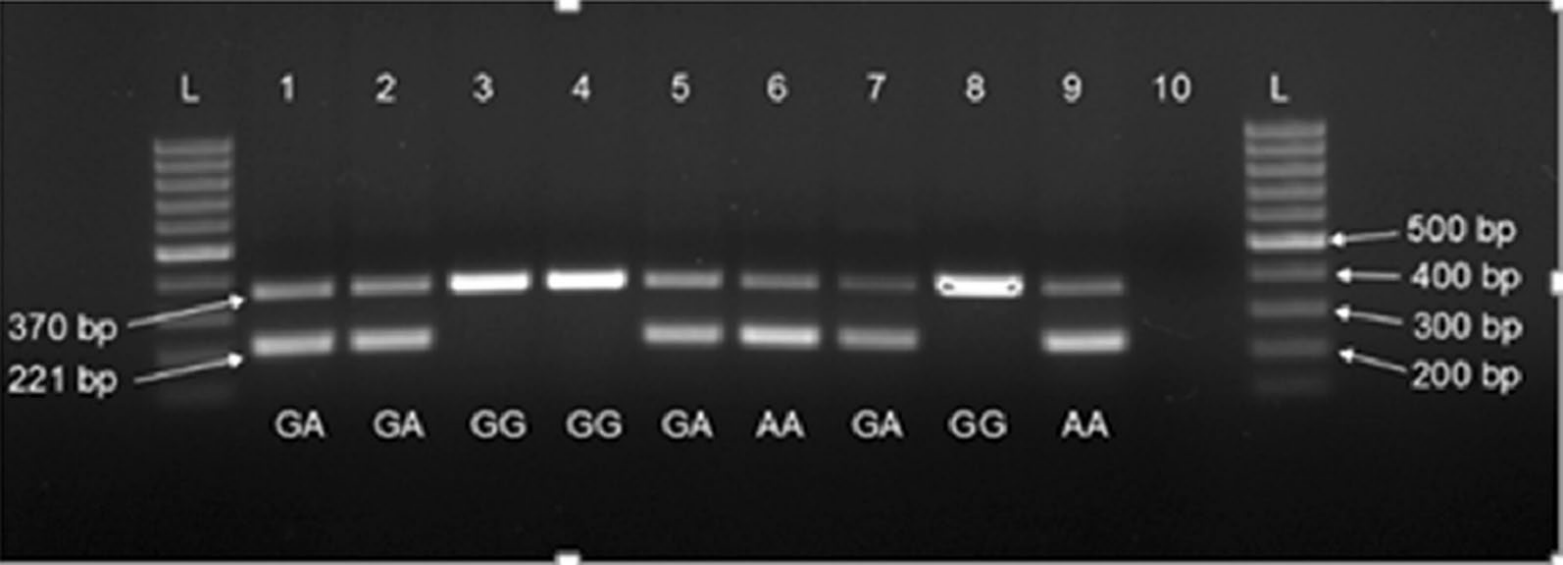
Verification of the duplication by PCR in animals with different genotypes at rs110675140. The upper band (370 bp) is an internal control that is amplified in all animals, while the lower band (221 bp) is the fragment that is amplified in animals carrying the duplication. GA, GG, and AA are the genotypes of the animals at rs110675140. *Lane 10* is a negative control, *L* is the 500 bp reference ladder

The presence and the orientation of the duplication were verified by PCR on DNA from 45 AI bulls and 112 randomly selected abattoir samples, and compared against rs110675140 genotypes. Bands that confirmed the duplication were observed for all 37 animals with genotype AA at rs110675140, and for 57 of the 58 animals with genotype GA (see Fig. 4 for examples). The duplication was absent in 60 of the 62 animals with genotype GG and very weak bands were observed for the two remaining GG animals. Thus, this duplication segregated almost perfectly with the alleles at rs110675140 in the NR population.

We aligned the entire sequence of the duplication against the human genome in order to search for conserved functional elements. The duplicated region overlapped the position of the alternative last exon 13 found in the predicted *GC* transcript variant X1 (XM_005208092.1), thus one possibility is that the presence of two copies of this exon may affect the level, stability or function of this transcript. A study that mapped regions enriched for acetylated lysine 27 on histone H3 (H3K27ac) via chromatin immunoprecipitation followed by high-throughput sequencing (ChIP-seq) showed that binding sites for enhancers were situated within the duplicated region (http://nagrp.ansci.iastate.edu/eric/epidb/rsem_expression.php#type1chipseq). Having two copies of this site could, in theory, affect transcription of *GC*.

The DNA sequence of the duplicated region showed remarkably little variation, since only 19 SNPs were detected in the entire ~12 kb region. None of the markers within or near the duplication were among the most significant for any of the traits.

### Putative function of the QTL

Since none of the markers in the coding regions were among the most significant, the causal polymorphism was expected to have a regulatory role. The *GC* gene is mainly transcribed in the liver [11]. Liver-specific *GC* expression in humans and rat was shown to be regulated through binding sites for the hepatocyte nuclear factor 1 (HNF1) that are situated 5′ of the transcription start site [34, 35], and through additional sites for the CCAAT/enhancer binding protein (C/EBP) that are located in intron 1 in humans [34]. First, we searched publicly available databases for putative HNF1 and C/EBP binding sites in the bovine sequence. The most likely HNF1 site was found between 88,739,350 and 88,739,364 bp on BTA6. This region is confirmed by the UCSC Genome Browser (https://genome.ucsc.edu), which reports a conserved HNF1 site 5′ of the human *GC* gene in a region that aligns to around 88,739,350 bp in the bovine sequence. This site is located near three of the most significant SNPs for CM1, i.e. rs109360654 at 88,739,045 bp (intron 1), rs108964685 at 88,739,413 bp (5′ region) and rs109541079 at 88,739,458 bp (5′ region). The most likely binding sites for C/EBP were found in the region between 88,727,907 and 88,727,918 bp, which is between rs109056048 at 88,727,480 bp and rs110675140 at 88,728,121 bp in intron 1. The human *GC* gene may also be regulated via two binding sites for the CCCTC-binding factor (zinc finger protein) (CTCF; http://ensembl.org). The sequences of these two sites align to regions that overlap the SNPs rs109056048 at 88,727,480 bp and rs109360654 at 88,739,045 bp, respectively. When searching the sequence that surrounds the most significant SNP for CM1 at 88,741,762 bp for putative transcription factor binding sites using the JASPAR database, putative binding sites for different members of the C/EBP family were detected in regions between 88,741,755 and 88,741,765 bp and between 88,741,764 and 88,741,773 bp, respectively.

### Characterization and quantification of *GC* transcripts

**Fig. 5:**
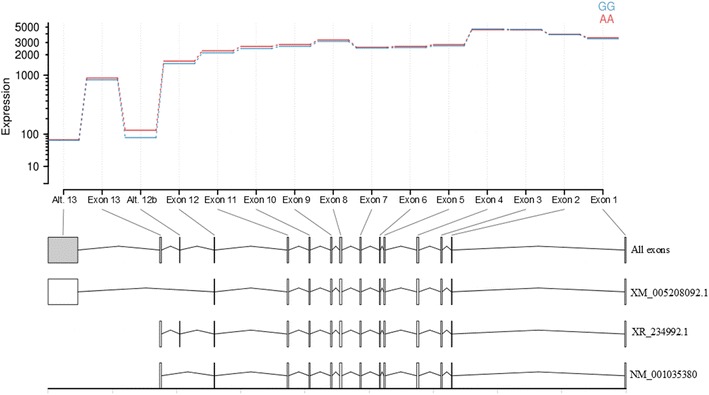
Expression of the differentially spliced *GC* mRNAs obtained from DEXSeq. *Blue* and *red* lines show expression of each exon in homozygous AA and GG animals at rs110675140, respectively. Exons included in the three transcripts are shown below. All: all identified exons. XM_005208092.1: exons in predicted transcript variant X1 (XM_005208092.1). XR_234992.1: exons in predicted transcript variant X2 (XR_234992.1), NM_001035380.2: exons in RefSeq transcript (NM_001035380.2)

**Fig. 6:**
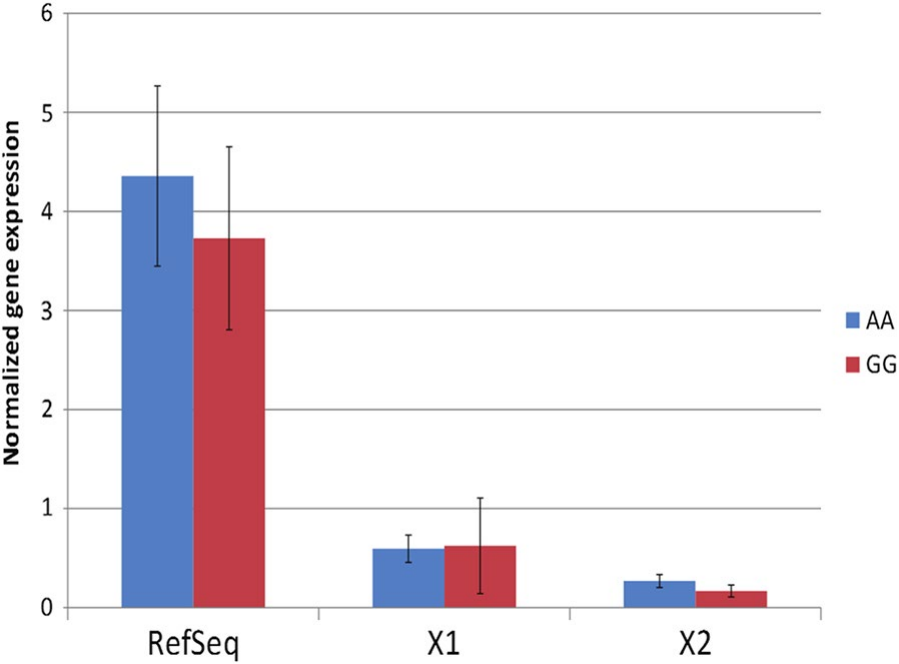
Transcript levels of homozygous AA and GG animals for the three detected *GC* transcripts obtained by RT-qPCR. AA and GG are the genotypes of the animals at rs110675140

Studies on the gene expression of *GC* in cattle show that it is predominantly expressed in liver, with no expression in the mammary gland (http://www.bovinegenome.org, http://nagrp.ansci.iastate.edu/eric/epidb/index.php, unpublished results in Norwegian Red cattle). To investigate if the QTL effect was due to differences in alternative splicing or expression levels of the *GC* mRNA, we tested liver samples from ten healthy individuals with different genotypes at rs110675140 (five AA and five GG animals). Results from RNAseq showed that all animals carried three transcripts with no significant difference between genotypes (not shown), i.e. the RefSeq transcript (NM_001035380.2) that consists of exons 1–13, the predicted transcript variant X1 (XM_005208092.1) in which exon 13 is replaced by an alternative last exon, and transcript variant X2 (XR_234992.1), which is similar to the RefSeq transcript except that it contains an additional small exon between exons 12 and 13 (Fig. 5). No other transcripts were detected. We detected several SNPs and short indels in the *GC* RNA sequences, but none of these segregated with the alleles at rs110675140. In the next step, the results from RNAseq were confirmed using RT-qPCR. Again, the same three transcripts were found in all tested animals (not shown), with no significant difference between genotypes (p > 0.05, Fig. 6). The level of the NM_001035380.2 transcript was higher than the levels of the less common X1 and X2 variants (p ≤ 0.001).

Although the DNA sequence showed that the alternative exon 13 was duplicated in animals with allele A at rs110675140, there was no evidence of expression of the duplication in the form of mRNA in the liver samples of any animal. Hence, we could not detect any effect of the duplication on the transcript levels under the examined conditions.

### Protein concentrations and vitamin D binding

The vitamin D-binding protein (DBP) encoded by the *GC* gene is the main carrier of vitamin D and its metabolites in serum. Vitamin D exists in two distinct forms: cutaneously-derived vitamin D3 and plant-derived vitamin D2. Both forms are metabolized in the liver into the major circulating metabolites, 25(OH)D3 and 25(OH)D2, respectively [36, 37]. Serum concentration of total 25(OH)D is considered to be a reliable marker for vitamin D status. In the kidney, 25(OH)D is further metabolized into the active hormone 1,25-dihydroxyvitamin D (see Additional file 3: Figure S1), with strictly regulated levels. We determined the concentrations of DBP, free vitamin D, DBP-bound vitamin D and related factors in serum, and tested for differences among animals with different genotypes at rs110675140. However, the GLM analyses showed no significant effect of the genotype at rs110675140 (p > 0.05) on any of the variables except for ALB (p = 0.04), with homozygous GG animals having higher ALB concentrations than homozygous AA animals [LSMEANS 36.2 vs. 34.1, (see Additional file 6: Table S4)].

Stage of lactation had a significant effect (p < 0.01) on the concentrations of DBP, 25(OH)D2, 25(OH)D3, free 25(OH)D2 and bioavailable 25(OH)D2. Concentrations of DBP and 25(OH)D3 increased while that of 25(OH)D2 decreased as lactation progressed. Concentrations of free 25(OH)D2 and bioavailable 25(OH)D2 were highest during the first month after calving and decreased thereafter. This is consistent with previous studies in humans [38, 39]. In cattle, 25(OH)D3 concentrations are reported to be associated with the pregnancy cycle and the lactation stage, with the highest 25(OH)D3 serum concentration observed during mid lactation [40]. Our study reports for the first time an association between the serum DBP concentrations and the lactation stage in dairy cattle. See (Additional file 6: Table S5) for more details.

### Evidence that supports *GC* as the positional and functional candidate gene for the QTL

Although high LD and long-range haplotypes in the QTL region complicate fine mapping and make it impossible to identify a causal polymorphism for the QTL, the results strongly support *GC* as the best positional candidate gene and agrees with results from other studies e.g. [6–10]. Sahana et al. [9] used whole-genome resequencing data to position the QTL for CM within a region that contains only two genes, *GC* and *NPFFR2*. They reported slightly different positions for this QTL in the two analyzed breeds, with the most significant SNPs located at 88.72 Mb (intron 1 of *GC*) in Nordic Red cattle, and at 88.97 Mb in Nordic Holstein. High LD in this region limited their ability to identify the causal polymorphism. The authors also tested for co-segregation with milk production traits in their Danish Holstein population and detected significant associations for kg milk, kg protein and kg fat between ~88.5 and 89.5 Mb [9], which is in agreement with our study.

In a follow-up study on Danish Holstein cattle, associations with CM were observed at three different locations in the region between 88.06 and 89.07 Mb [41]. One QTL overlapped with the *deoxycytidine kinase* (*DCK*) gene, the second overlapped with the *solute carrier family 4, sodium bicarbonate co*-*transporter, and member 4* (*SLC4A4*) gene, and the last QTL overlapped with the *neuropeptide FF receptor 2* (*NPFFR2*) gene. No significant QTL within or near the *GC* gene were detected in Danish Holstein, which may indicate that different polymorphisms affect susceptibility to CM in Holstein as compared to Nordic and Norwegian Red breeds. However, the LD between the SNPs in the third QTL (i.e. at *NPFFR2*) and the most significant SNPs in our study was high (not shown), and indicates that this third QTL in Danish Holstein is the same as the QTL detected in Nordic Red and Norwegian Red.

In addition, Goddard et al. [10] reported *GC* as the gene that underlies a QTL for milk production in an Australian Holstein population. Again, in this study, the high LD in the QTL region made it difficult to pinpoint a causal polymorphism. However, they identified the most significant SNPs at 88,741,762 and 88,743,767 bp depending on the methods. These SNPs are the most significant and third most significant SNPs for CM1 in our study, and among the most significant for the remaining traits.

*Group-specific component* encodes DBP, which has multiple important biological roles in the immune system and host defense. DBP is the major plasma protein carrier of vitamin D3 and its metabolites. Vitamin D3 has a function as a cytokine that is generated to protect the host against microbial invaders [42]. During an infection, both human and bovine monocytes and macrophages express an enzyme that converts vitamin D into its biologically active form [43–45], which then regulates the expression of the vitamin D responsive genes [46]. Recent studies indicate that vitamin D signaling has important roles in the immune function and infectious disease resistance in cattle [44, 45, 47], and DBP was found to be a serum biomarker during subclinical mycobacterial infections in cattle [48].

Vitamin D-binding protein was also suggested to be a precursor of the macrophage activating factor Gc-MAF [49]. Activated macrophages develop the capacity for specialized tasks such as chemotaxis, phagocytosis and the lysis of intracellular parasites, as well as the destruction of tumor cells [50]. Furthermore, DBP was shown to increase monocyte and neutrophil chemotaxis to complement component C5a (C5a)-derived peptides [51, 52].

DBP may have a direct effect on milk production. Goddard et al. [10] suggested that this effect could be mediated through the number and activity of mammary epithelial cells, since vitamin D restricts growth and differentiation of these cells in tissue culture [53–55]. Vitamin D also has a key function in maintaining serum levels of calcium by stimulating intestinal calcium absorption, mobilization of calcium from bone and stimulating the renal reabsorption at the onset of lactation, when large amounts of calcium are secreted into the milk and colostrum [56]. The association that we detected between serum DBP and vitamin D concentrations with lactation stage also suggests a role of DBP and vitamin D in regulating milk production in dairy cattle.

Hence, the QTL could either affect host immune response to pathogens and indirectly decrease milk production in diseased animals, or the main effect could be on increased milk production with a secondary effect on mastitis, for instance due to lack of energy for immune defense or strain on the teat canal and sphincter muscle from increased volume at each milking. Alternatively, the mechanisms behind milk production and immune response could be affected more or less independently by the same underlying polymorphism, or by multiple polymorphisms in high LD with each other.

In this study, we did not succeed in identifying the functional variant that is responsible for the QTL. A limitation of our study was the choice of the animals used for RNA expression and protein analyses. Ideally, such analyses should use samples from cows with different genotypes during a mastitis infection. However, sampling such material with uniform quality and in sufficiently large amounts for proper statistical analysis poses a rather complicated logistics challenge, at least in Norway where the farms are small and situated far apart, and would need a long period of time. This was beyond the duration and resources of this project, and analyses were therefore restricted to material that was readily available. RNA expression was analyzed in liver samples, since the *GC* gene is most highly expressed in this tissue [11]. However, most of the available samples were from healthy bulls, and the QTL seems to show functional significance only in females or during an infection. Protein analyses were performed in serum samples from cows, but these were also healthy animals. A more successful strategy to identify the nature of the QTL might be to investigate RNA expression and protein levels in cows with mastitis.

## Conclusions

Association analysis of full sequence data mapped a highly significant QTL that affects both clinical mastitis in early lactation and milk production to a region within or immediately upstream of the *GC* gene. We also detected a duplication downstream of the *GC* gene that segregated with the alleles of this QTL. The duplication covered the alternative last exon of the *GC* gene and might contain binding sites for enhancers. No differences neither in *GC* transcript levels nor in protein levels and binding were detected under the examined conditions, but our data did not permit any final conclusions to be drawn on differences in transcription or protein binding during an infection. Further functional studies including both healthy and infected animals are needed to reveal the causative variants that underlie this QTL.

## Additional files

**Additional file 1: Table S1.** SNP genotype, pregnancy status, age, lactation number and lactation status for the cows used for analyses of protein concentrations and ligand binding.

**Additional file 2: Table S2.** Primer sequences used for the reverse transcription qPCR (RT-qPCR).

**Additional file 3: Figure S1.** Further description of the procedure for estimating DBP protein levels. Detailed description of the protocol for estimating the DBP levels in serum from 50 NR cows. Figure S1 gives a schematic explanation of vitamin D3 metabolism.

**Additional file 4: Table S3.** Results for single marker association analyses of seven mastitis traits and five milk traits on BTA6 markers from the Illumina BovineHD BeadChip. rs numbers, positions in basepairs, −log_10_(p value), SNP effects and alleles for all markers on BTA6 on the Illumina BovineHD BeadChip. The file contains twelve sheets, one for each trait.

**Additional file 5: Table S4.** Results for single marker association analyses of CM1, CM4, CM6, kg milk, kg protein and kg fat on imputed full sequence data. Positions in basepairs, rs numbers, −log_10_(p value), minor allele frequency, SNP effects, alleles, putative consequences of the variants on the protein sequence and nearest gene for the imputed full sequence SNPs. The file contains six sheets, one for each tested trait.

**Additional file 6: Table S5.** Results for General Linear Model (GLM) analyses of effect of rs110675140 genotype on DBP, ALB, 25(OH)D2, 25(OH)D3 and total 25(OH)D; free 25(OH)D2, 25(OH)D3 and total 25(OH)D; bioavailable (i.e., free and ALB-bound) 25(OH)D2, 25(OH)D3 and total 25(OH)D; DBP-bound total 25(OH)D; and ALB-bound total 25(OH)D serum concentrations. Detailed results of GLM analyses of genotype effect on several serum protein levels in 50 NR cows.
