# Supplementary material for: Fine mapping of a QTL on bovine chromosome 6 using imputed full sequence data suggests a key role for the group-specific component (GC) gene in clinical mastitis and milk production
Source: Genet Sel Evol. 2016 Oct 19;48:79. doi: 10.1186/s12711-016-0257-2 (PMC5072345; doi:10.1186/s12711-016-0257-2)
Supplement: Supplementary file 2 — Additional file 2: Table S2. Primer sequences used for the reverse transcription qPCR (RT-qPCR). [file 12711_2016_257_MOESM2_ESM.docx]

**Additional File 2.**

List of primers used for reverse transcription-quantitative PCR (RT-qPCR).

| Gene symbol, transcript name and accession no. | Primers (5’🡪3’) | Amplicon (bp) |
| --- | --- | --- |
| GC, RefSeq, NM_001035380.2 | ACCATCTTAAACCTCAAGCTGG ACAAATCATCACGCTTAACATCA | 151 |
| GC, X1, XM_005208092.1 | TCTTAATTTCTTGAAGGTGATGC  TCTGAACTTCAATTGCCAGCT | 172 |
| GC, X2, XR_234992.1 | TGCATGTGTACCATCTTAAGTTAT  GGTGTTGCTGAGTTTAGGCC | 110 |
