# Supplementary material for: Fine mapping of a QTL on bovine chromosome 6 using imputed full sequence data suggests a key role for the group-specific component (GC) gene in clinical mastitis and milk production
Source: Genet Sel Evol. 2016 Oct 19;48:79. doi: 10.1186/s12711-016-0257-2 (PMC5072345; doi:10.1186/s12711-016-0257-2)
Supplement: Supplementary file 3 — Additional file 3: Figure S1. Further description of the procedure for estimating DBP protein levels. Detailed description of the protocol for estimating the DBP levels in serum from 50 NR cows. Figure S1 gives a schematic explanation of vitamin D3 metabolism. [file 12711_2016_257_MOESM3_ESM.docx]

**Additional File 3.**

**Further details for estimation of levels of serum vitamin D.**

50 µl of internal standard working solution (100 ng/ml 25-OHD2-d3 and 25-OHD3-d3) was added to 100 µl serum, calibrators or internal quality control samples. The samples were allowed to equilibrate for at least 10 minutes before the addition of 300 µl precipitation solution (2% NH3 in ACN). The samples were left at -20°C for 25 minutes to obtain satisfactory precipitation. The supernatant was transferred to a HybridSPE 96-well plate (Supelco, Bellefonte, PA, USA) for extraction. The solvent was removed by N2 (25 min, 70 °C) and the samples were reconstituted in 100 µl 50/50 MeOH/H_2_O (v/v). LC-MS/MS analysis was performed using a 1290 UHPLC system (Agilent, Santa Clara, CA, USA) coupled to a 6490 tandem mass spectrometer (Agilent). Of the sample, 20 µl were injected onto a 2.1x50 mm high strength silica-pentafluorophenyl (HSS-PFP) column (Waters, Milford MA, USA) heated to 40°C. Mobile phase A consisted of 0.2% FA in water, while mobile phase B consisted of 0.2% FA in MeOH. A linear gradient from 72% B to 75% B in 1.5 min at a flow rate of 0.4 ml/min was used to separate and elute the analytes. Detection was performed with positive electrospray ionization and multiple reaction monitoring. Collision energies of 20V and 10V were used for fragmentation of 25(OH)D_2_/25(OH)D_2_-d3 and 25-OHD3/25-OHD3-d3, respectively. The ion transitions monitored for quantification of 25-OHD2, 25-OHD2-d3, 25-OHD3 and 25-OHD3-d3 were 395.3→269.1, 398.2→272.2, 383.3→257.2 and 386.8→257.0. Additionally, one or two ion transitions (qualifier ions) were monitored for the analytes and internal standards, respectively.

Kidney: 24-hydroxylase (CYP24A1)


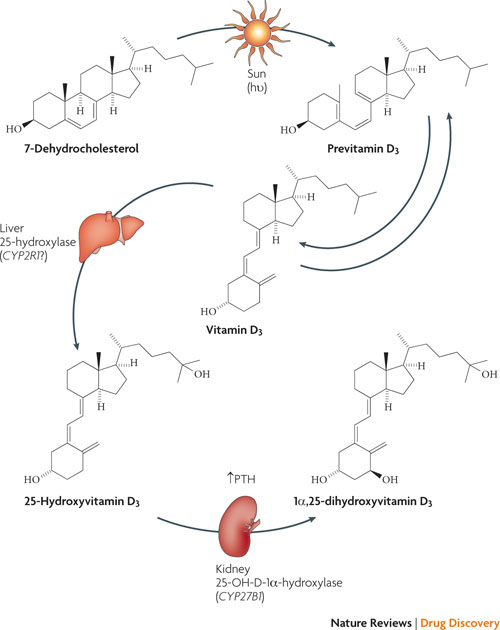


Liver: 25-hydroxylase (CYP2R1)


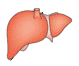

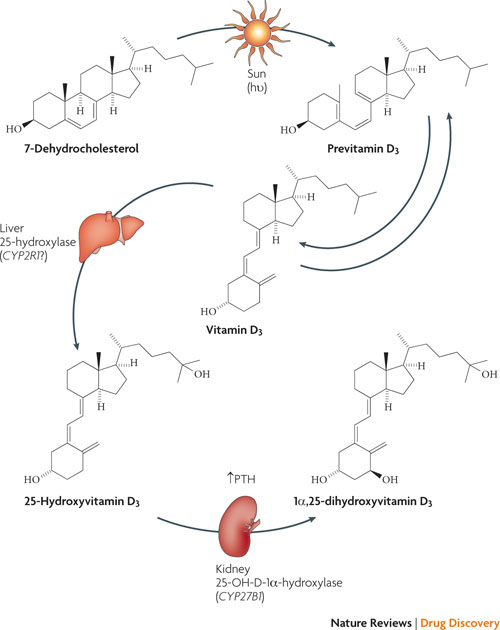


C3 epimerization


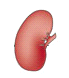

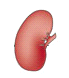

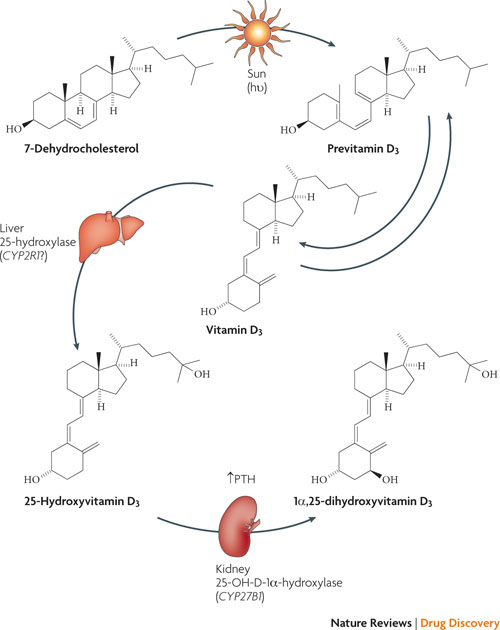

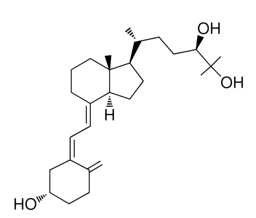

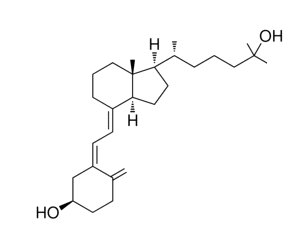


**25-Hydroxyvitamin D_3_**

**1α, 25-dihydroxyvitamin D_3_**

**Vitamin D_3_**

Kidney: 1α-hydroxylase (CYP27B1)

**24(R), 25-dihydroxyvitamin D_3_**

**3-epi-25-Hydroxyvitamin D_3_**

**Figure S1.** Metabolism of Vitamin D3, Metabolism of Vitamin D2 is corresponding to metabolism of vitamin D3
